# The *Z*′ = 12 superstructure of Λ-cobalt(III) sepulchrate trinitrate governed by C—H⋯O hydrogen bonds

**DOI:** 10.1107/S2052520616005503

**Published:** 2016-05-26

**Authors:** Somnath Dey, Andreas Schönleber, Swastik Mondal, Siriyara Jagannatha Prathapa, Sander van Smaalen, Finn Krebs Larsen

**Affiliations:** a Laboratory of Crystallography, University of Bayreuth, Bayreuth, Germany; b Department of Chemistry, Aarhus University, Aarhus, Denmark

**Keywords:** *Z*′ superstructure, hydrogen bonding, superspace, commensurately modulated structure

## Abstract

Λ-Cobalt(III) sepulchrate trinitrate crystallizes in *P*6_3_22 with *Z* = 2 (*Z*′ = 1/6) at room temperature. Slabs perpendicular to the hexagonal axis comprise molecules Co(sepulchrate) alternating with nitrate groups *A* and *B*. Coordinated by six sepulchrate molecules, highly disordered nitrate groups *C* are accommodated between the slabs. Here we report the fully ordered, low-temperature crystal structure of Co(sep)(NO_3_)_3_. It is found to be a high-*Z*′ structure with *Z*′ = 12 of the 12-fold  superstructure with monoclinic symmetry *P*2_1_ (**c** unique). Correlations between structural parameters are effectively removed by refinements within the superspace approach. Superstructure formation is governed by a densification of the packing in conjunction with ordering of nitrate group *C*, the latter assuming different orientations for each of the *Z*′ = 12 independent copies in the superstructure. The Co(sep) moiety exhibits small structural variations over its 12 independent copies, while orientations of nitrate groups *A* and *B* vary less than the orientations of the nitrate group *C* do. Molecular packing in the superstructure is found to be determined by short C—H⋯H—C contacts, with H⋯H distances of 2.2–2.3 Å, and by short C—H⋯O contacts, with H⋯O distances down to 2.2 Å. These contacts presumably represent weak C—H⋯O hydrogen bonds, but in any case they prevent further densification of the structure and strengthening of weak N—H⋯O hydrogen bonds with observed H⋯O distances of 2.4–2.6 Å.

## Abstract 2

The 12-fold superstructure at *T* = 95 K is described as a commensurately modulated structure in superspace. Crystal packing in the *Z*′ = 12 superstructure is determined by short C—H⋯O hydrogen bonds.

## 1. Introduction   

**Figure 1:**
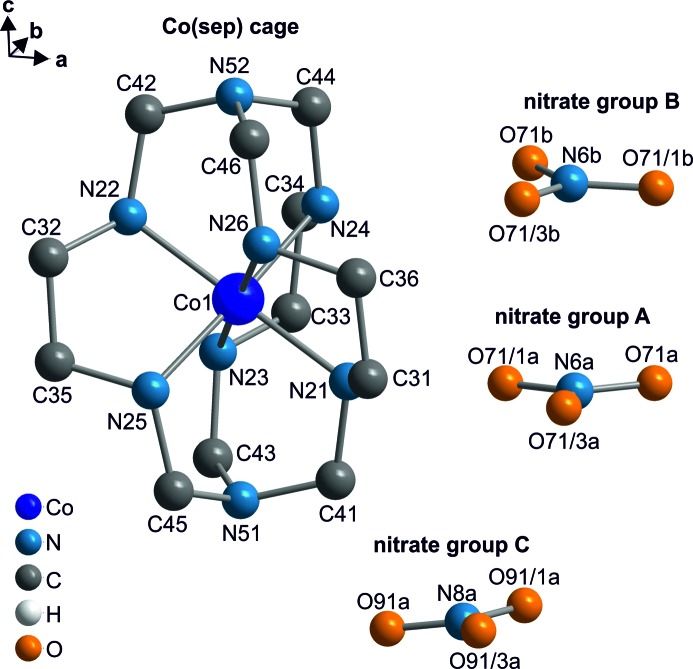
Molecular structure of of Λ-cobalt(III) sepulchrate trinitrate along with the atomic numbering scheme. H atoms have been omitted for clarity

Macrobicyclic metal cage complexes are templating agents for the synthesis of silicates (Hondow *et al.*, 2012 ▸) and zeolites (Garcia *et al.*, 2001 ▸). These compounds also serve as catalysts enhancing electrochemical processes (Ritzert *et al.*, 2013 ▸), as a photosensitizer for the production of hydrogen from water (Pina *et al.*, 1985 ▸) and as reducing agents in the field of bio­electrochemistry (Bernhardt *et al.*, 2006 ▸). The crystal structures of these compounds provide useful information because applications of these compounds are correlated to their thermodynamic stability, stereochemistry, size and the nature of coordination inside the cages, and the strong and weak interaction of the cages with the functional groups (Gahan & Harrowfield, 2015 ▸).


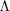
-Cobalt(III) sepulchrate trinitrate, Co(sep)(NO_3_)_3_ [IUPAC name 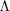-(1,3,6,8,10,13,16,19-octaazabicyclo[6,6,6]eicosane)cobalt(III) trinitrate] is a sepulchrate cage complex. The sepulchrate moiety consists of a Co^3+^ cation sixfold coordinated by N atoms of the six amine groups (N_lig_ = N21, N22, N23, N24, N25, N26 in Fig. 1 ▸) of the sepulchrate molecule. The amine groups act as donors of lone pairs of electrons to the 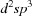 hybridized Co^3+^ cation conforming a distorted octahedron. The macrobicyclic cage is completed by three ethylene groups (C_en_ = C31, C36; C33, C34; C32, C35), six apical C atoms (C_ap_ = C41, C42, C43, C44, C45, C46) and two apical N atoms (N_ap_ = N51, N52 in Fig. 1 ▸).

The compound crystallizes in hexagonal symmetry with space group 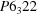 at room temperature (phase I) (Dubicki *et al.*, 1980 ▸; Schönleber *et al.*, 2010 ▸). The Co(sep) cage and two symmetry-related nitrate groups (nitrate groups *A* and *B*) are centred on threefold rotation axes. They have been reported to be connected *via* N—H⋯O hydrogen bonds (Dubiciki *et al.*, 1984 ▸). The third nitrate group (nitrate group *C*) is slightly displaced from the origin, exhibiting a sixfold orientational disorder (Schönleber *et al.*, 2010 ▸).

A phase transition of Co(sep)(NO_3_)_3_ has been found to occur at *T*_1_ = 133 K; spectroscopic methods have indicated that below *T*_1_ the crystals develop a domain structure, as it may appear due to a lowering of its symmetry (Dubiciki *et al.*, 1984 ▸). Single-crystal neutron diffraction experiments have revealed satellite reflections below *T*_1_. Their temperature dependence has indicated two further phase transitions, at *T*_2_ = 107 K and *T*_3_ = 98 K, respectively (Larsen *et al.*, 1988 ▸).

We have performed temperature-dependent single-crystal X-ray diffraction studies on Co(sep)(NO_3_)_3_, which confirmed the existence of different phases at *T* = 115 K (phase II), 100 K (phase III) and 95 K (phase IV; Dey *et al.*, 2016 ▸). The two intermediate phases II and III possess incommensurately modulated crystal structures (Larsen *et al.*, 1988 ▸).

The present X-ray diffraction experiments reveal the low-temperature phase (phase IV) to be a lock-in phase with a 12-fold 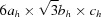 superstructure of the room-temperature hexagonal structure. The phase transition from phase III to phase IV is identified as an incommensurate-to-commensurate transition accompanied by major changes of the modulation wave. Specifically, the disordered nitrate group *C* becomes fully ordered in phase IV, assuming different orientations at different sites within the 12-fold supercell.

The order as well as the accompanying atomic displacements and molecular distortions are described by modulation functions within the superspace approach (van Smaalen, 2012 ▸; Schönleber, 2011 ▸; Janssen *et al.*, 2007 ▸).

The origin of the modulation is discussed in terms of molecular conformations and intermolecular interactions, including the role of hydrogen bonding.

## 2. Experimental   

### 2.1. Diffraction experiment and data integration   

Single crystals of Co(sep)(NO_3_)_3_ were synthesized by the research team of Alan M. Sargeson (Creaser *et al.*, 1982 ▸). The crystals are stable in air at ambient conditions. Single-crystal X-ray diffraction experiments were performed at beamline D3, Hasylab DESY, Hamburg, employing radiation of a wavelength of 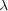 = 0.50917 Å. The sample was mounted on a four-circle diffractometer in Euler geometry, and diffraction intensities were measured by a MAR-CCD area detector. A data set complete up to a resolution of 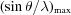 = 0.838 Å^−1^ was measured by 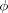 and ω scans with several exposure times and different offsets of the detector, always maintaining a crystal-to-detector distance of 220 mm and using scans of 1° per image (Table 1 ▸).

Data processing has been performed with the software *EVAL*15 (Schreurs *et al.*, 2010 ▸). Inspection of the frames revealed that main reflections are surrounded by satellite reflections of first order. Bragg reflections have been indexed using five integers 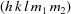 on a primitive hexagonal lattice and with modulation wavevectors
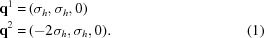
Refinement of the hexagonal orientation matrix and the component of the modulation wavevector resulted in 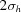 = 0.16726 (10) = 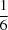 + 0.00059 (10), suggesting an incommensurate modulation.

**Figure 2:**
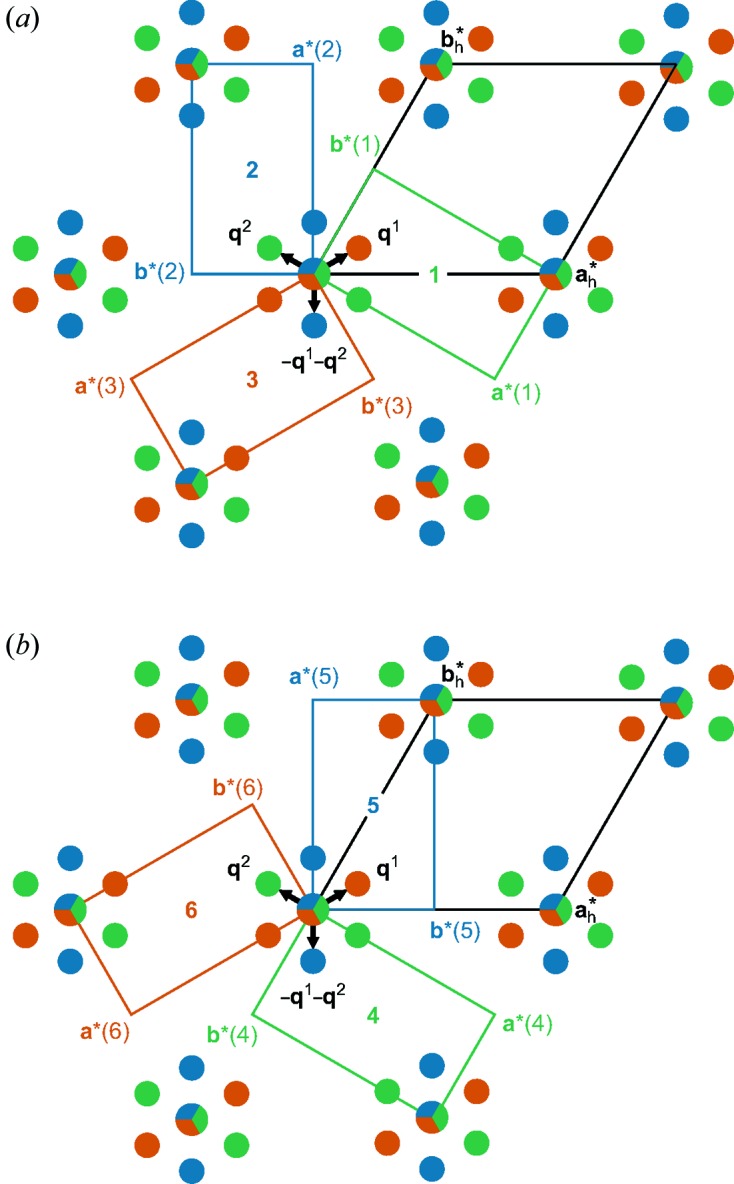
Schematic drawing of the diffraction pattern at *T* = 95 K exhibiting hexagonal to monoclinic sixfold twinning. (*a*) **q** ^2^, **q** ^1^ + **q** ^2^ and **q** ^1^ belong to the green (1), blue (2) and red (3) orthorhombic or monoclinic domains, respectively. (*b*) Monoclinic twin domains 4, and 5 and 6 are generated by applying twofold rotation **a** axes parallel to the twin domains 1, 2 and 3, respectively. The assignment of the **q**-vectors to the twin domains has been chosen consistent with *International Tables for Crystallography*, Vols. A and C (Arnold, 2006 ▸; Janssen *et al.*, 2006 ▸). The reconstructed reciprocal layer *hk*2 is given as Fig. S1 in the supporting information.

However, diffraction images exhibit diffraction maxima that appear as overlapping Bragg reflections from different domains rather than single peaks. Therefore, we have used an alternative indexing of the diffraction pattern on the *C*-centred 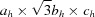 orthohexagonal unit cell. In the case of orthorhombic symmetry, each main reflection is composed of three different, nearly coincident Bragg reflections from the three domains, while satellite reflections have contributions from only a single domain (Fig. 2 ▸ *a*). With respect to the orthohexagonal unit cell the satellite reflections are indexed by 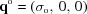 with 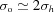.

In the case of monoclinic symmetry, each main reflection is composed of six different, nearly coincident Bragg reflections from the six domains; satellite reflections now have contributions from two domains (Fig. 2 ▸). Each domain of monoclinic symmetry is modulated by a single modulation wavevector
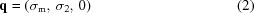
with 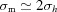 and 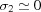.

Refinement with *EVAL*15 of the orthorhombic lattice parameters and the orientations of the six lattices resulted in a very small orthorhombic lattice distortion together with 
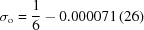
. Further lowering of the symmetry to monoclinic resulted in a lattice distortion given by the angle 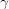 = 89.9943 (3)° and 
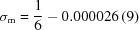
. This value is much closer to the commensurate value 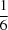 than the value obtained with the hexagonal lattice and it differs from 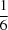 by less than three standard uncertainties. The discrepancies can be explained by the fact that the twinned refinement determines the position of the modulation wavevector with respect to the reciprocal lattice in its own domain, whereas the two-dimensional modulation on the hexagonal lattice determines the average satellite position with respect to the average lattice of all domains. A non-zero value for 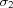 leads to instable refinements and a non-significant value for this parameter. Together, we take these refinements as experimental evidence for the commensurability of the modulation in phase IV.

The severe overlap of Bragg reflections from different domains prevents the determination of integrated intensities of individual reflections. Instead, accurate intensities can be obtained for each group of overlapping reflections. Furthermore, available software for absorption correction cannot simultaneously handle twinned and incommensurate data. Therefore, integrated intensities have been determined within the hexagonal setting, employing the two modulation wavevectors from equation (1). Integrated intensities have been obtained for main reflections, 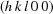 and satellite reflections of first order, 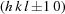, 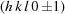 and 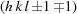, employing *EVAL*15. The absorption correction was determined by *SADABS* (Sheldrick, 2008 ▸). Throughout these calculations, monoclinic point symmetry 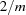 (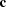 unique) has been used. This is the minimum symmetry of the diffraction pattern in the case of twin domains of unequal volume fractions. Only for equal volume fractions of the domains can the point symmetry become higher, *e.g.* 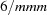 in the case of all volume fractions being equal.

After data reduction, the dataset was split into one subset of main reflections (common to all twin domains) and three subsets of satellites reflections (for the three twin domains 1, 2 and 3; Fig. 2 ▸ *a*; twin matrices are given in Table 1 ▸). The indices were transformed from 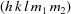 to 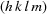 without changing *h*, *k* or *l*, and with 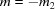 for the twin domain 1, 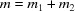 for twin domain 2 and 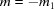 for twin domain 3. The reflection indices were then transformed to the *C*-centred orthohexagonal setting (Arnold, 2006 ▸), using the software *JANA*2006 (Petricek *et al.*, 2014 ▸). The three additional monoclinic twin domains 4, 5 and 6 were described by applying a twofold rotation along 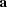 to the domains 1, 2 and 3, respectively. In this way, satellite reflections of each of the three pairs of domains coincide with each other according to: 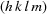 of domain 1 coincides with 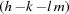 of domain 4; main reflections of all six domains coincide. The ratio of the average intensities 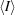 between main and satellite reflections is approximately 60:1, while the average significance 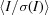 has a ratio of 4:1 between the main and satellite reflections.

### 2.2. Structure solution and refinement   

A model for the 12-fold superstructure of phase IV of Co(sep)(NO_3_)_3_ at *T* = 95 K has been developed within the superspace approach (van Smaalen, 2012 ▸). The basic structure is nearly identical to the crystal structure of phase I, but it has been described on the *C*-centred orthohexagonal unit cell (Table 1 ▸). Consideration of diffraction symmetry (Table 1 ▸) and reflection conditions has led to the space group 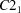 (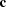-unique) for the basic structure, where the monoclinic unique axis is equal to the hexagonal axis of phase I. This space group is a non-standard setting of 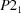, No. 4 in *International Tables for Crystallography*, Vol. A. The superspace group has been found as 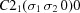 with 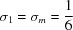 and 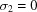. This superspace group is a non-standard setting of 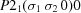, No. 4.1.2.1 in Stokes *et al.* (2011 ▸) and van Smaalen *et al.* (2013 ▸).

Formally, charge flipping does not apply to diffraction data from crystals twinned by (pseudo-)merohedry. Nevertheless, *SUPERFLIP* (Palatinus & Chapuis, 2007 ▸) applied to the present data allowed us to determine the positions of the Co atom along with initial values for its modulation parameters. The positions of the other non-H atoms were derived from the atomic positions of phase I (Schönleber *et al.*, 2010 ▸), employing *JANA*2006 for the application of group–subgroup relations following the reduction of symmetry from 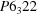 to 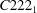 to 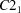. The low monoclinic symmetry of the pseudo-hexagonal basic structure is responsible for large correlations between structural parameters. Therefore, restraints on interatomic distances and bond angles of the Co(sep) cage have been introduced in accordance with the crystal structure of phase I (Schönleber *et al.*, 2010 ▸). The nitrate groups have been described as planar molecules with point symmetry 32, leaving the N—O distance as a single refinable parameter. One nitrate molecule is placed in two independent positions corresponding to the nitrate groups *A* and *B*. A second nitrate molecule is used for nitrate group *C*. Each independent position is defined by three translational and three rotational parameters. The modulations of the nitrate groups were initially described by small values for the amplitudes of the first-order harmonic waves for modulation of translations and rotations. Displacement modulations of the other non-H atoms have been described by a single harmonic wave with initially small values for the parameters, except cobalt, for which the parameters obtained by *SUPERFLIP* have been used. H atoms were added to all ligand N atoms (N_lig_), and all ethylene (C_en_) and apical (C_ap_) C atoms by a riding model, keeping tetrahedral geometries with distances *d*(N—H) = 0.87 Å and *d*(C—H) = 0.96 Å. Atomic displacement parameters (ADPs) of H atoms follow from 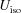
(H) = 1.2 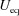
(parent atom). Structure refinements of this (3 + 1)-dimensional superspace model have been performed with *JANA*2006. Employing isotropic ADPs and the incommensurate approximation, the refinement converged at 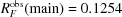 and 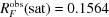 for main and satellite reflections, respectively.

In the next step, anisotropic ADPs were introduced for the non-H atoms in the Co(sep) cage and TLS parameters (Schomaker & Trueblood, 1968 ▸) were applied to the nitrate groups. Refinement resulted in a much improved fit to the main reflections with 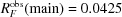 and 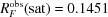, and a large reduction of the features in the difference Fourier map, *viz* 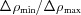 reduced from −2.26/10.84 to −1.02/2.19 e Å^−3^. Subsequently, first-order harmonic waves have been introduced for the modulation of ADPs of non-H atoms of the Co(sep) cage and for modulation of the TLS parameters of the nitrate groups. Refinement resulted in an improved fit to the satellite reflections with 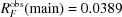 and 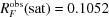.

The next step involved the introduction of first-order harmonic waves for the third-order Gram–Charlier parameters of the Co atom, while the basic third-order Gram–Charlier parameters were constrained to remain zero (Li *et al.*, 2011 ▸). The fit to the diffraction data improved again at 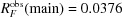 and 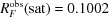. More importantly, ADPs of the ten atoms in the Co(sep) cage, which were previously non-positive definite, become positive definite by this procedure.

In the final model constraints of the riding model on the fractional coordinates and modulation parameters of the H atoms attached to the six N_lig_ atoms were replaced by restraints on distances and angles. Restraint parameters are *d*(N—H) = 0.87 ± 0.02 Å and ∠(H—N—Co) = ∠(H—N—C3) = ∠(H—N—C4) = 109.47 ± 1°. Refinement resulted in an again improved fit, with 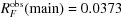 and 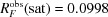, and much reduced features in the difference Fourier map (Table 1 ▸). The plot of 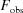 *versus* 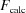 shows an excellent match between these quantities (Fig. S2 in the supporting information).

**Figure 3:**
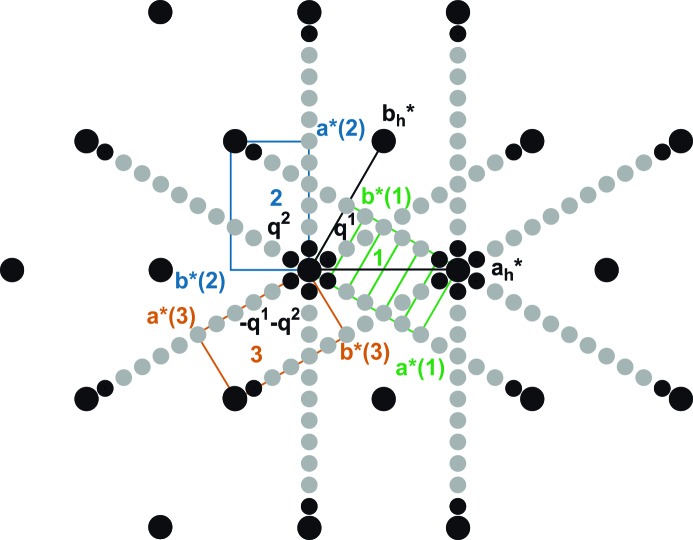
Schematic drawing of the diffraction pattern at *T* = 95 K. Positions are indicated of all possible commensurate satellite reflections. Black circles represent observed reflections while grey circles denote reflection positions unobserved in the present experiment. The sixfold supercell corresponding to the green domain in Fig. 2 ▸ is highlighted by thin lines.

The commensurability of the modulation requires the selection of the correct value of the initial phase, 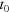, of the modulation wave (van Smaalen, 2012 ▸). For the 12-fold superstructure, the modulation wave is sampled at 12 equidistant points. Since only first-order satellite reflections have been observed (Fig. 3 ▸), this implies that different 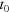 as well as the incommensurate model provide fits to the diffraction data of nearly equal quality. Table 2 ▸ gives partial *R* values for three different values of 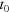. We believe that the minor differences in *R* values are not sufficient to uniquely determine 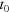. Nevertheless, 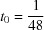 has slightly higher *R* values and represents a supercell structure with triclinic symmetry, while 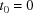 gives the lowest *R* values and corresponds to the highest supercell symmetry with space group 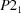 (a model with the same symmetry is obtained for 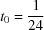). Evidence for lowering the symmetry to triclinic thus is lacking and we have chosen the monoclinic model with 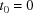 for further analysis.

The relatively high partial 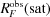 value for satellite reflections (Table 2 ▸) can be explained by rather weak reflections, as it is expressed by the magnitudes of partial *R* values for satellite reflections for the averaged standard uncertainty over intensity 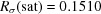 and for averaging of equivalent reflections 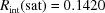. Further evidence for the final structure model has been obtained by establishing that the following variations of the model did not improve the fit to the diffraction data:

(i) Treatment of nitrate group *A* and nitrate group *B* as different molecules leads to large correlations in the positions of their O atoms without any significant changes in *R* values.
(ii) The Co(sep) cage was refined without distance and angle restraints and individual atoms were refined for the nitrate groups. This model results in improved statistical parameters of refinement [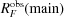 = 0.0335 and 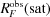 = 0.0975], but the ADPs of four C atoms are non-positive definite and the geometries of the nitrate groups are outside the physically possible range.
(iii) Since the symmetry is non-centrosymmetric, inversion twinning was introduced for all six twin domains. The refinement resulted in insignificant values of the inversion twin volumes.
(iv) Second-order harmonic waves were added for displacive modulation of the atoms, but the resulting modulation functions showed ripples indicating fitting of the noise in the data.
(v) Isotropic secondary extinction correction resulted in a negative extinction parameter.

## 3. Discussion   

### 3.1. Variation of molecular conformations for *Z*′ = 12   

**Figure 4:**
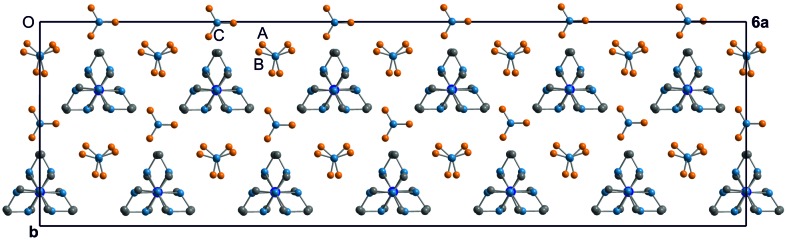
Unit cell of the superstructure projected onto (0 0 1). Atoms with −0.05 < *z* < 0.45 are shown (one layer; *cf.* Fig. 5 ▸). H atoms are not shown.

The crystal structure of the low-temperature phase (phase IV) of Co(sep)(NO_3_)_3_ is a high-*Z*′ structure. Only a few crystal structures feature more independent molecules in the unit cell than the presently observed *Z*′ = 12 [for example, the amino acid l-tryptophan with *Z*′ = 16 (Görbitz *et al.*, 2012 ▸); see also the review article by Steed & Steed (2015 ▸) and references therein, as well as the high *Z*′ structure database by Steed (2016 ▸)]. High-*Z*′ structures usually suffer from severe correlations between parameters in structure refinements, and restrictions on the parameters beyond symmetry restrictions are necessary. An elegant way of imposing nonsymmetry restrictions is the superspace approach (Schönleber, 2011 ▸; van Smaalen, 2012 ▸; Pinheiro & Abakumov, 2015 ▸). Parameters are separated into a relatively small subset of atomic coordinates describing the basic structure [one molecule Co(sep) and three nitrate ions] and parameters describing deviations from the basic structure. For Co(sep)(NO_3_)_3_ we have observed satellite reflections in the X-ray diffraction of first order only (Fig. 3 ▸), which implies that the superstructure is described by a single harmonic modulation function for each independent parameter of the basic structure; higher harmonics cannot be determined (see the end of §2.2). Deviations from the basic structure are small indeed, as is easily seen by consideration of a plot of the superstructure, showing the 12 crystallographically independent but nearly indistinguishable copies of the molecule (Figs. 4 ▸ and 5 ▸).

The superspace approach reduces the number of independent parameters dramatically compared with the 12-fold supercell. Nevertheless, the monoclinic superspace group of phase IV does not capture the pseudo-trigonal symmetry of the Co(sep) molecule, and further restrictions have turned out to be necessary. We have chosen to use rather narrow restraints on bond lengths and bond angles (§2.2). They are justified in hindsight by the good fit to the diffraction data of the thus refined structure model (Tables 1 ▸ and 2 ▸).

Unsurprisingly, the 12 crystallographically independent Co(sep) molecules in the 12-fold supercell feature similar values for each bond length and each bond angle. The variations are most favourably analysed by *t*-plots (van Smaalen, 2012 ▸), which provide the continuous dependence of each structural parameter on phase *t* of the modulation wave. Values observed in the 12-fold superstructure with *t*_0_ = 0 are the values *t* = *n*/12 for *n* = 0,…, 11. Fig. 6 ▸ provides a *t*-plot of the distances between the Co atom and the 12 C atoms of the same molecule. It shows that these distances (not restrained in the refinement) have but minor variations between the 12 molecules. The (small) elongation of one Co—C bond is compensated for by shortening another Co—C bond, in complete agreement with the behaviour of other modulated compounds. Presently, distances towards the three apical C atoms at the top of the molecule (Nos. 10–12 in Fig. 6 ▸) are longer than average simultaneously with the distances towards the three apical C atoms at the bottom of the molecule being shorter than average (Nos. 7–9). This reflects the largest modulation amplitude of Co being along **c** (Table S1 in the supporting information). Angles within the Co(N_lig_)_6_ octahedron deviate more from their ideal values than in the structure of phase I (Schönleber *et al.*, 2010 ▸), but their variation with *t* is again very small (see the supporting information). Bond lengths and bond angles of all 12 independent Co(sep) molecules thus are similar to each other. The average molecule in phase IV has a slightly more distorted geometry than the structure of the molecule within the high-symmetry phase (Schönleber *et al.*, 2010 ▸) as well as in other filled sepulchrate complexes (Bacchi *et al.*, 1993 ▸; Bacchi *et al.*, 1996 ▸; Gahan & Harrowfield, 2015 ▸).

Nitrate groups have been restricted to be flat with O—N—O angles of 120° and three equal N—O bond lengths (Fig. 1 ▸). Nitrate groups *A* and *B* have been described by a common molecule with a refined N—O bond length of 1.237 (6) Å. The N—O bond in nitrate group *C* is shorter with *d*(N—O) = 1.160 (7) Å. Major differences between the different copies of the nitrate groups *A*, *B* and *C* are the orientations of these planar molecular anions (Fig. 7 ▸). We believe this part of the modulation to be at the origin of the complicated superstructure, as described in the next section.

### 3.2. Competing intermolecular interactions   

**Figure 5:**
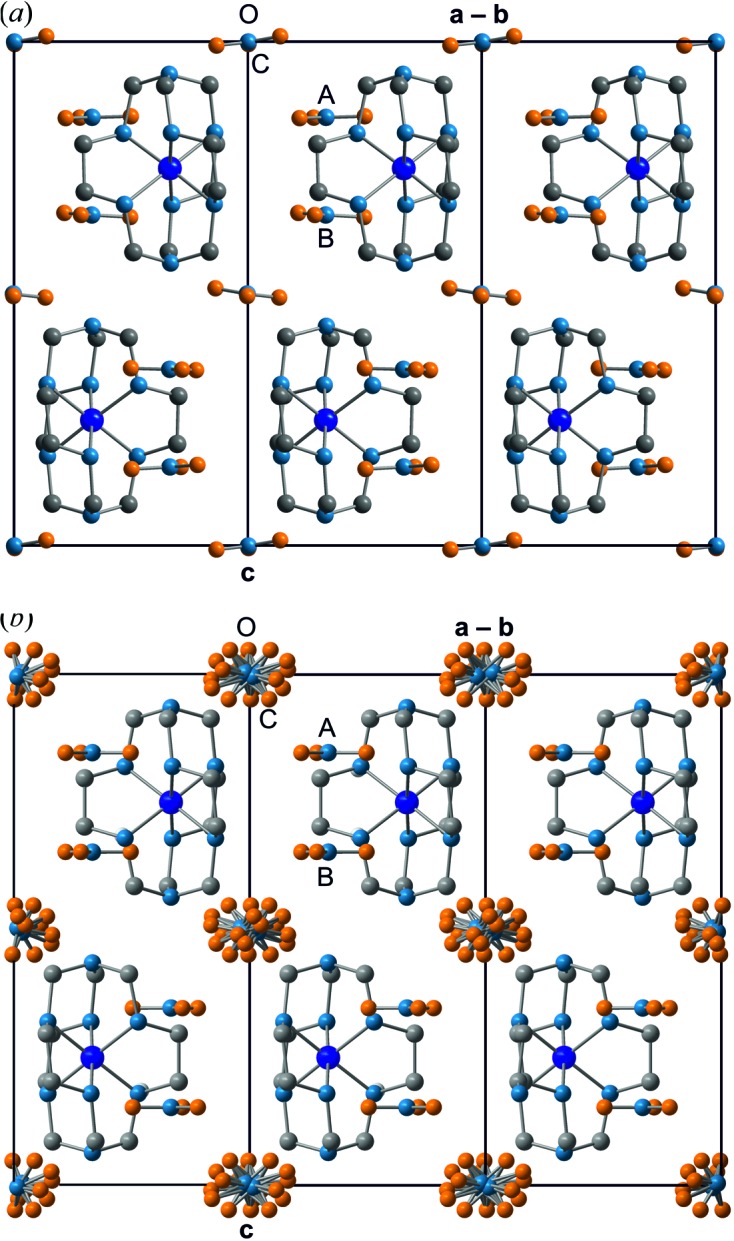
(*a*) The basic structure at *T* = 95 K (phase IV) projected onto (1 1 0) and (*b*) the disordered phase at room temperature (phase I; Schönleber *et al.*, 2010 ▸). H atoms are not shown.

**Figure 6:**
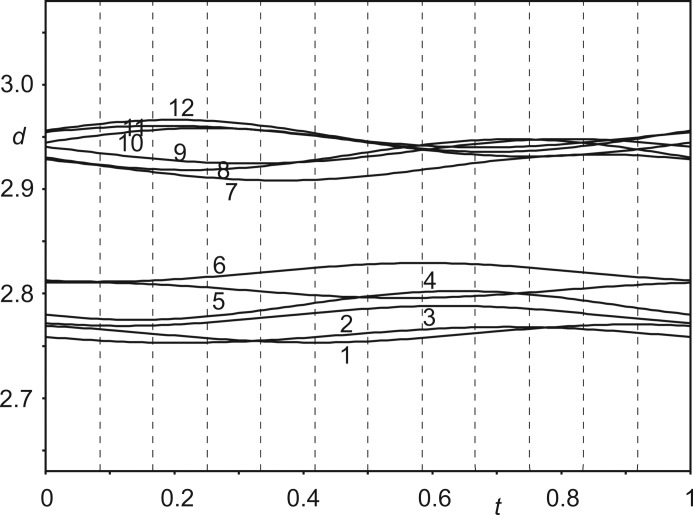
*t*-Plot of interatomic distances *d* (Å) between the Co atom and the C atoms of the same molecule. Vertical, dashed lines indicate *t* values corresponding to the distances in the 12-fold supercell. The 12 interatomic contacts are (*cf.* Fig. 1 ▸): 1 = Co1—C36, 2 = Co1—C34, 3 = Co1—C33, 4 = Co1—C35, 5 = Co1—C31, 6 = Co1—C32, 7 = Co1—C45, 8 = Co1—C43, 9 = Co1—C41, 10 = Co1—C44, 11 = Co1—C42 and 12 = Co1—C46.

**Figure 7:**
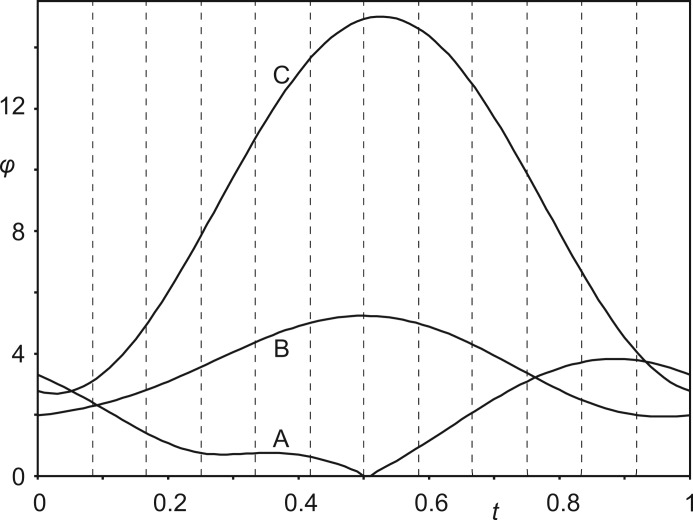
*t*-Plot of the angles φ (°) between the (**a**,**b**)-plane and the molecular planes of the nitrate groups *A*, *B* and *C*. Vertical dashed lines indicate *t* values corresponding to the angles in the 12-fold supercell.

In phase I, the nitrate groups *A* and *B* are parallel to the (**a**, **b**)-plane. In the present structure (phase IV), the planes of the 12 nitrate groups *A* and the 12 nitrate groups *B* make different angles with the (**a**, **b**)-plane, with values between 0 and 5° (Fig. 7 ▸). These small rotations are the result of optimizing the interactions between nitrate and sepulchrate moieties (see below). Nitrate group *C* is highly disordered in phase I (Fig. 5 ▸ *b*; Schönleber *et al.*, 2010 ▸). The increased packing density at lower temperatures forces each nitrate group *C* into a single orientation. Orientations of the 12 crystallographically independent nitrate groups *C* exhibit a much larger variation than orientations of nitrate groups *A* and *B* (Fig. 7 ▸), again apparently the result of optimizing intermolecular interactions.

**Figure 8:**
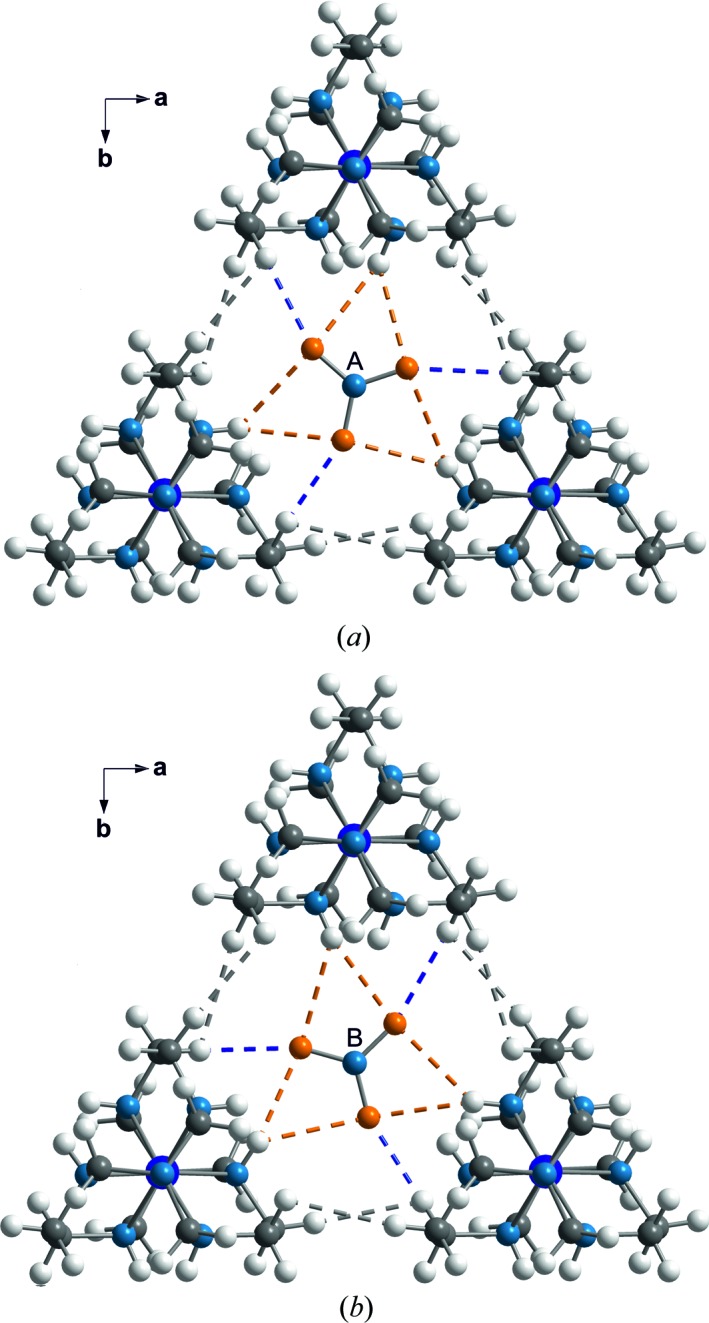
Environments of the nitrate groups *A* and *B* in a view along (0 0 1). Dashed lines indicate N—H⋯O hydrogen bonds (orange), C—H⋯O hydrogen bonds (blue) and short C—H⋯H—C contacts between participating sepulchrate cages (grey). (*a*) Nitrate group *A* at *z* = 0.15; (*b*) nitrate group *B* at *z* = 0.34. Co(sep) cages are centred at *z* = 1/4 (*cf.* Fig. 5 ▸).

**Figure 9:**
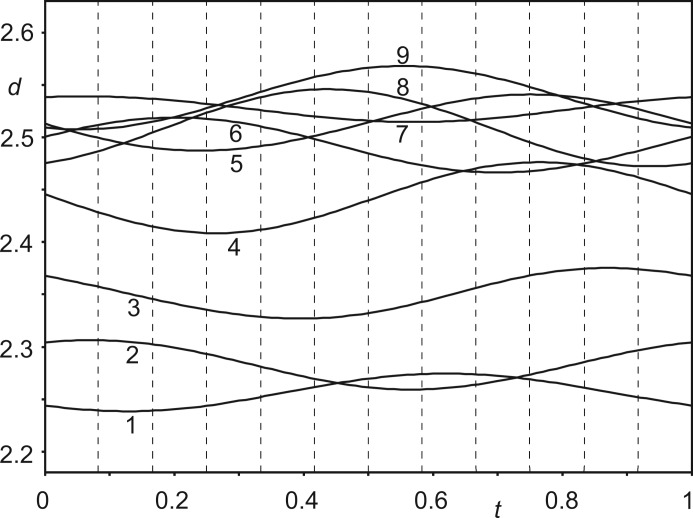
*t*-Plot of interatomic distances *d* (Å) between the O atom of nitrate group *A* and H atoms of the sepulchate cage. Vertical, dashed lines indicate *t* values corresponding to the distances in the 12-fold supercell. Six N—H⋯O contacts (4–9) and three C—H⋯O contacts (1–3) are shown (*cf.* Fig. 1 ▸): 1 = O71/3*a*⋯H2*c*35, 2 = O71*a*⋯H2*c*31, 3 = O71/1*a*⋯H2*c*33, 4 = O71/3*a*⋯H1*n*21, 5 = O71/1*a*⋯H1*n*21, 6 = O71*a*⋯H1*n*25, 7 = O71/1*a*⋯H1*n*25, 8 = O71/3*a*⋯H1*n*23, and 9 = O71*a*⋯H1*n*23. A similar plot for nitrate group *B* is given as Fig. S9 in the supporting information.

Nitrate groups *A* and *B* form N—H⋯O hydrogen bonds with the sepulchrate cages (Fig. 8 ▸). Distances H⋯O vary between ∼ 2.4 and 2.6 Å for the six H⋯O interactions about each nitrate group, but each contact exhibits but a small variation between the 12 independent nitrate groups *A* and *B*, as shown by the *t*-plot of these distances (4–9 in Fig. 9 ▸). This implies that they are weak hydrogen bonds (Jeffrey, 1997 ▸), which are hardly affected by the modulation. One would expect the molecules to close in, in order to strengthen the N—H⋯O hydrogen bonds. However, this is prevented by actually the shortest interatomic distances between molecules.

Nitrate groups *A* and *B* are involved in C—H⋯O interactions, which are shorter than the N—H⋯O hydrogen bonds (Fig. 9 ▸). As short as 2.2 Å, these H⋯O distances are at the lower side of the reported C—H⋯O hydrogen bonds (Desiraju, 1991 ▸; Desiraju *et al.*, 1993 ▸; Steiner & Desiraju, 1998 ▸; Desiraju & Steiner, 2001 ▸; Munshi & Row, 2005 ▸). It can thus be conjectured that even closer separations of these atoms would take them onto the repulsion part of the interaction curve, which would be energetically unfavourable. C—H⋯O interactions rather than N—H⋯O hydrogen bonds thus determine the packing in Co(sep)(NO_3_)_3_.

**Figure 10:**
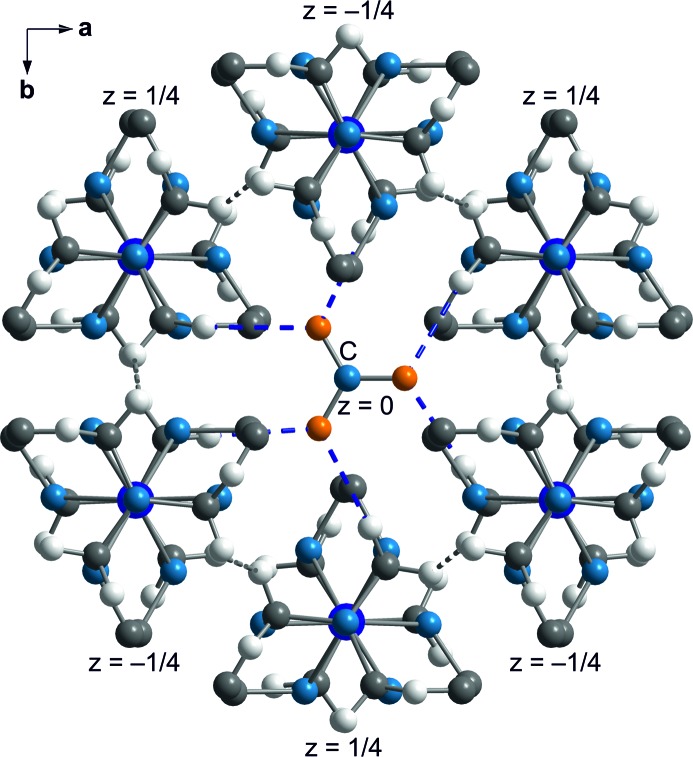
Projection onto (0 0 1) of nitrate group *C* at *z* = 0 and its six coordinating Co(sep) cages at *z* = ±1/4, as indicated. The shortest intermolecular contacts are C—H⋯O hydrogen bonds (blue dashed lines) and C—H⋯H—C contacts (grey dashed lines).

**Figure 11:**
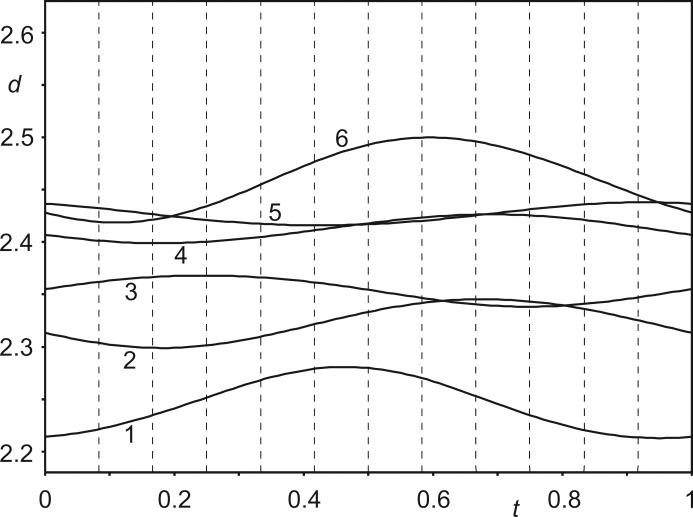
*t*-Plot of interatomic distances *d* (Å) between the O atom of nitrate group *C* and H atoms of the sepulchate cage. Vertical, dashed lines indicate *t* values corresponding to the distances in the 12-fold supercell. Six C—H⋯O contacts are shown (*cf.* Fig. 1 ▸): 1 = O91*a*⋯H2*c*42, 2 = O91/1*a*⋯H2*c*44, 3 = O91/3*a*⋯H2*c*43, 4 = O91/1*a*⋯H2*c*45, 5 = O91/3*a*⋯H2*c*46, and 6 = O91*a*⋯H2*c*41.

**Figure 12:**
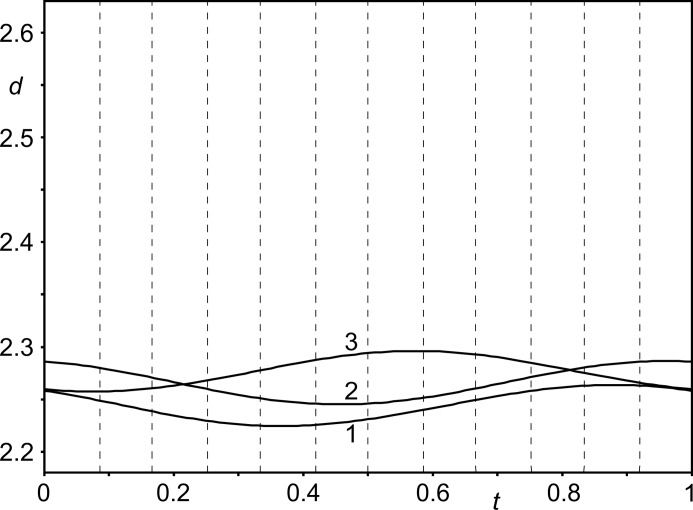
*t*-Plot of interatomic distances *d* (Å) between the H atoms attached to apical C atoms of neighbouring sepulchrate molecules. Vertical, dashed lines indicate *t* values corresponding to the distances in the 12-fold supercell. 1 = H1*c*42⋯H1*c*43; 2 = H1*c*41⋯H1*c*44; 3 = H1*c*45⋯H1*c*46.

The shortest contacts between nitrate group *C* and the sepulchrate cage are between the O atoms (atoms O9) and H atoms connected to the apical C atoms (atoms H2*c*41 through to H2*c*46) (Fig. 10 ▸). The range of H⋯O distances of ∼ 2.2–2.5 Å is similar to the distances of the C—H⋯O contacts of nitrate groups *A* and *B* (Fig. 11 ▸). Finally, short intermolecular interatomic distances are found between H atoms attached to apical C atoms on neighbouring sepulchrate molecules (Fig. 12 ▸). Again, these contacts prevent a denser packing of the molecules.

## 4. Conclusions   

The 12-fold superstructure of Λ-cobalt(III) sepulchrate trinitrate at *T* = 95 K has successfully been described within the superspace approach. The superspace approach effectively removes correlations between crystallographically independent parameters of this high-*Z*′ structure with *Z*′ = 12.

A major difference between phase I – stable at ambient conditions – and phase IV – stable below 98 K – is the order of the nitrate group *C* (Fig. 4 ▸). At room temperature nitrate group *C* exhibits at least sixfold orientational disorder, while in phase IV this nitrate group is fully ordered in 12 different orientations in the 12-fold supercell (Fig. 7 ▸).

The origin of the modulation is argued to lie in the avoidance of repulsive interactions rather than hydrogen bonding between neighbouring molecules. The shortest interatomic contacts are C—H⋯O weak hydrogen bonds (Figs. 9 ▸ and 11 ▸) and C—H⋯H—C intermolecular H-atom contacts (Fig. 12 ▸). Intermolecular N—H⋯O hydrogen bonds do exist (Fig. 9 ▸), but they are long and weak, apparently prevented from full development by the short intermolecular contacts described above.

## Supplementary Material

Crystal structure: contains datablock(s) global, I. DOI: 10.1107/S2052520616005503/dk5043sup1.cif

Structure factors: contains datablock(s) I. DOI: 10.1107/S2052520616005503/dk5043Isup2.hkl

Supporting figures and tables. DOI: 10.1107/S2052520616005503/dk5043sup3.pdf

11752ErO4oI

CCDC reference: 1472105

## Figures and Tables

**Table 1:** Experimental and crystallographic data

Crystal data	
Chemical formula	Co(C_12_H_30_N_8_)(NO_3_)_3_
*M*_r_	531.4
Crystal system, superspace group	Monoclinic *c*-unique, *C*2_1_(σ_1_σ_2_0)0
Temperature (K)	95
Wavevector	**q** = 0.166667a*
*a*, *b*, *c* (Å)	8.4283 (1), 14.5984 (1), 15.7057 (1)
γ (°)	89.9943 (3)
*V* (Å^3^)	1932.42 (3)
*Z*	4
Radiation type	Synchrotron, λ = 0.50917 Å
μ (mm^−1^)	0.39
Crystal size (mm)	0.25 × 0.13 × 0.10
Commensurate section	*t*_0_ = 0
Supercell	6*a* × *b* × *c*
Supercell space group	*P*2_1_
	
Diffraction data	
(sin θ/λ)_max_ (Å^−1^)	0.83777
Δφ, Δω (°)	1
Exposure time (s)	4, 10, 80
Absorption correction	Empirical, multiscan
Criterion of observability	*I* > 3σ(*I*)
Unique reflections	
All (obs/all)	19 292/59 443
Main (obs/all)	7916/8468
Satellites (obs/all)	11 376/50 975
*R*_int_ (obs/all)	0.0183/0.0225
	
Refinement	
GoF (obs/all)	2.11/1.32
*R*_obs_/*wR*_all_	0.0493/0.0662
No. of parameters	865
H-atom treatment	mixed
Twin volumes (1, 2)	0.163 (2), 0.1480 (11)
(3, 4)	0.1845 (11), 0.1384 (11)
(5, 6)	0.1731 (11), 0.1932 (11)
Twin matrices (2)	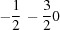 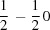 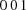
(3)	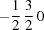 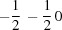 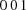
(4)	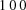 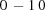 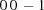
(5)	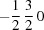 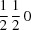 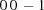
(6)	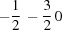 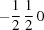 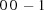
Δρ_min_, Δρ_max_ (e Å^−3^)	−0.60, 0.58

**Table 2:** Statistical parameters (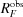 and 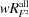) of commensurate refinements of the final model with different values of the phase *t*_0_

	*t*_0_ = 0	*t*_0_ = 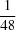	*t*_0_ = 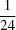
Supercell space group	*P*2_1_	*P*1	*P*2_1_
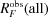	0.0493	0.0496	0.0493
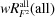	0.0662	0.0665	0.0662
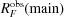	0.0374	0.0378	0.0374
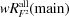	0.0490	0.0496	0.0490
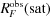	0.0998	0.1000	0.0998
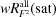	0.1250	0.1252	0.1250
Δρ_min_ (e Å^−3^)	−0.58	−0.61	−0.58
Δρ_max_ (e Å^−3^)	0.60	0.59	0.60
