# Supplementary material for: The Z′ = 12 superstructure of Λ-cobalt(III) sepulchrate trinitrate governed by C—H⋯O hydrogen bonds
Source: Acta Crystallogr B Struct Sci Cryst Eng Mater. 2016 May 26;72(Pt 3):372–80. doi: 10.1107/S2052520616005503 (PMC4886617; doi:10.1107/S2052520616005503)
Supplement: Supplementary file 3 [file b-72-00372-sup3.pdf]

Supplementary material

The $Z' = 12$ superstructure of Λ-cobalt(III) sepulchrate trinitrate governed by C–H···O hydrogen bonds

Somnath Dey,[a] Andreas Schönleber,[a*] Swastik Mondal,[a] Siriyara Jagannatha Prathapa,[a] Sander van Smaalen,[a] and Finn Krebs Larsen[b]

[a]*Laboratory of Crystallography, University of Bayreuth, Bayreuth, Germany,*
and
[b]*Department of Chemistry, Aarhus University, Aarhus, Denmark.*

E-mail: andreas.schoenleber@uni-bayreuth.de

Contents

Supplementary Figures S1–S12 and supplementary Table S1.

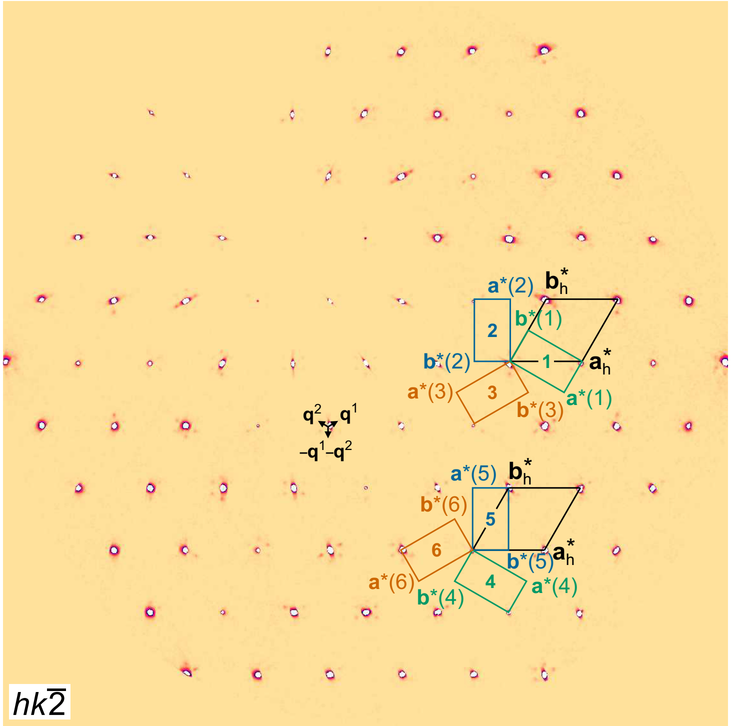

Figure S1: Reconstruction of the reciprocal lattice plane $hk\overline{2}$ with the hexagonal unit cell and the ones for the monoclinic six-fold twinning, exhibiting strong main and weak satellite reflections.

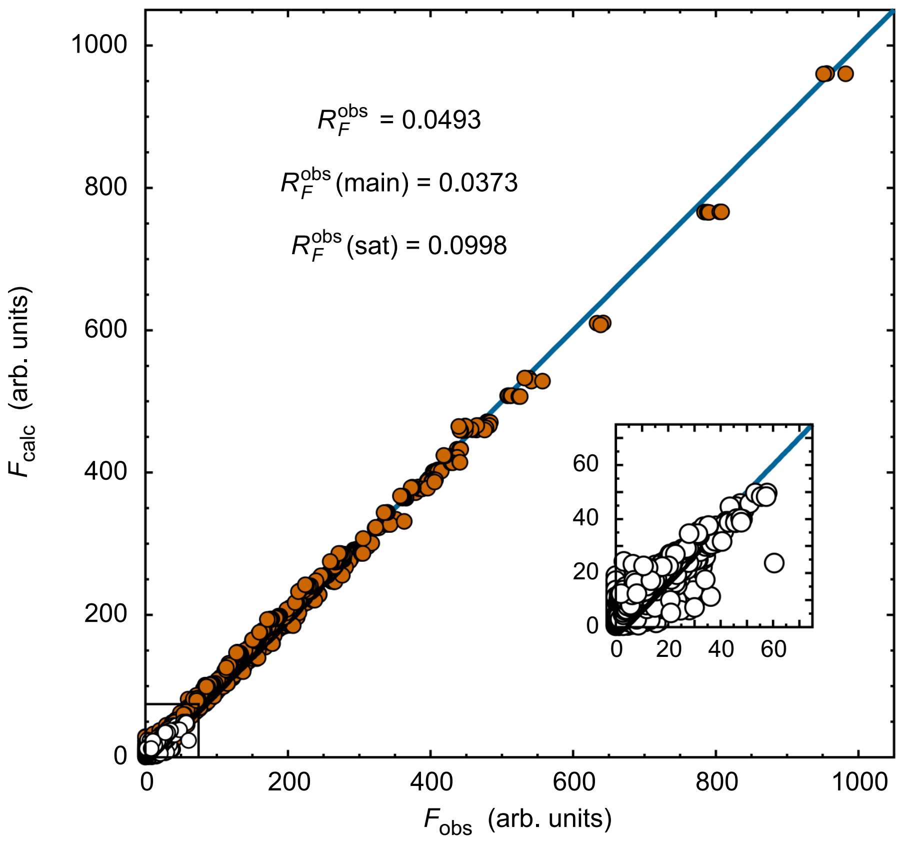

Figure S2: Plot of F_{obs} *vs.* F_{calc} of the 8468 main (orange circles) and 50975 satellite reflections (white circles) applied in the refinement of the final structural model. In the inset only the satellite reflections are shown.

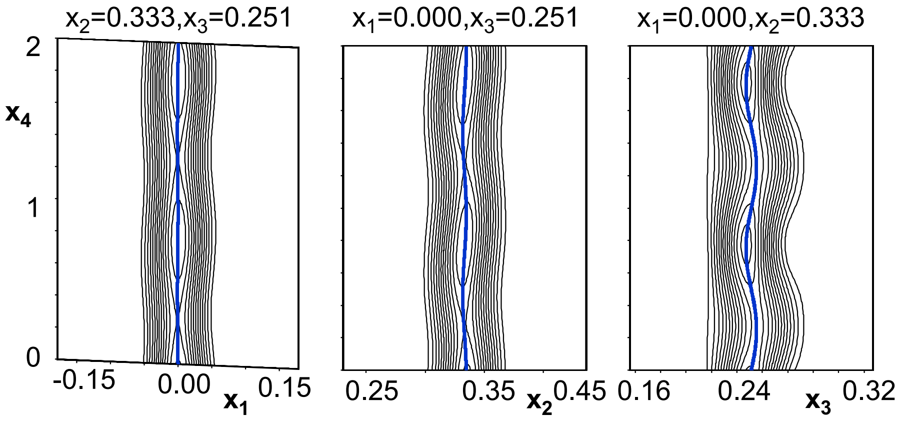

Figure S3: Sections (x_1,x_4), (x_2,x_4) and (x_3,x_4) centered at the position of the cobalt atom of the Fourier map of Co(sep)$(NO_3)_3$ at $T = 95$ K. They reveal a smooth modulation described by harmonic waves of first order. Contour lines of equal density are at intervals of 1.0 e/Å^3. The thick blue curve represents the refined modulation function. The width of each panel is 3 Å.

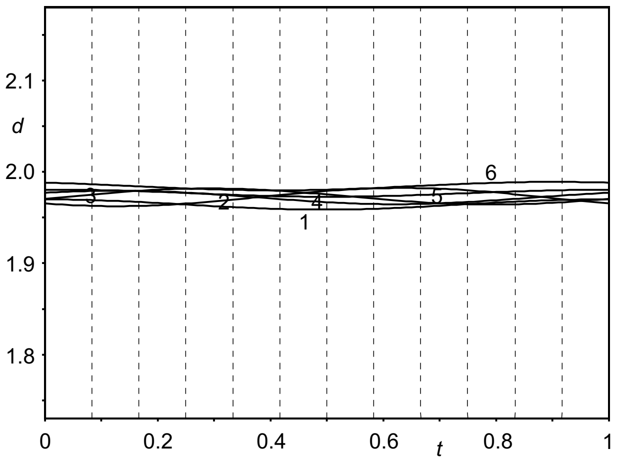

Figure S4: t-Plot of interatomic distances d (Å) between the cobalt atom and the six coordinating nitrogen atoms N_{lig}. Vertical, dashed lines indicate t values corresponding to the distances in the 12-fold supercell.

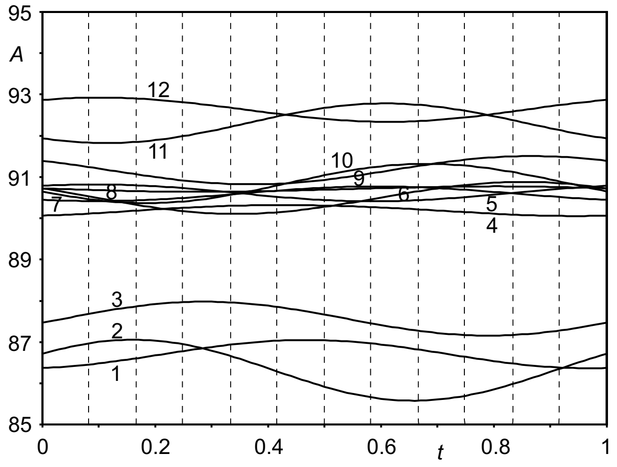

Figure S5: t-Plot of angles N_{lig}–Co–N_{lig} (deg), involving the six coordinating nitrogen atoms N_{lig}. The variation with t of individual angles is smaller than the average distortion of the CoN_6 octahedron. 1=N21–Co1–N26, 2=N22–Co1–N25, 3=N23–Co1–N24, 4=N22–Co1–N23, 5=N21–Co1–N24, 6=N22–Co1–N26, 7=N25–Co1–N26, 8=N23–Co1–N25, 9=N24–Co1–N26, 10=N22–Co1–N24, 11=N21–Co1–N25, and 12=N21–Co1–N23.

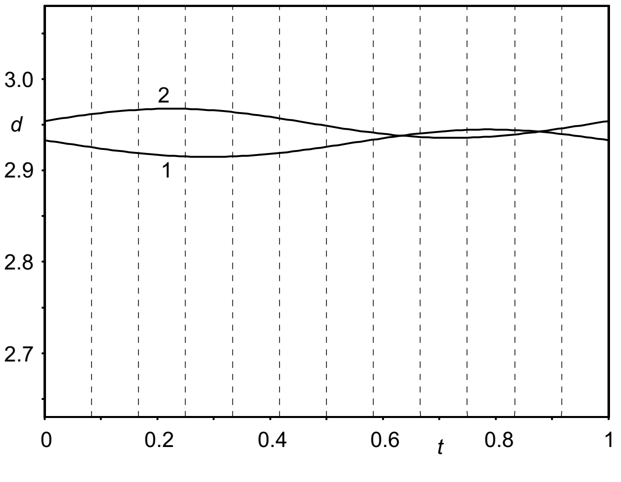

Figure S6: t-Plot of interatomic distances d (Å) between the cobalt atom and the two capping nitrogen atoms N51 and N52.

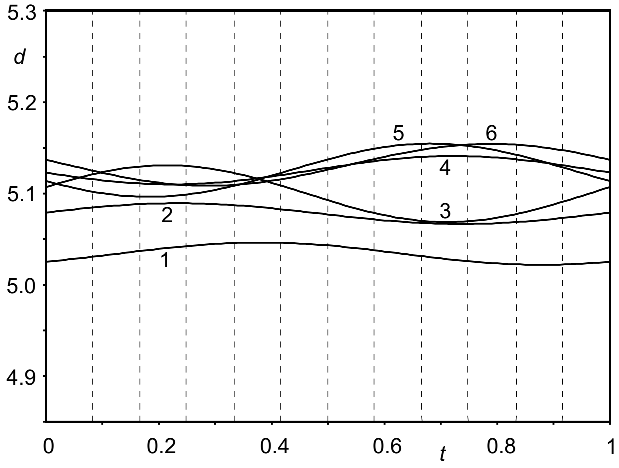

Figure S7: t-Plot of interatomic distances d (Å) between the cobalt atom and the nitrogen atoms of nitrate groups A and B. One cobalt atom is coordinated by three nitrate groups A (3, 5 and 6) and three nitrate groups B (1, 2 and 4).

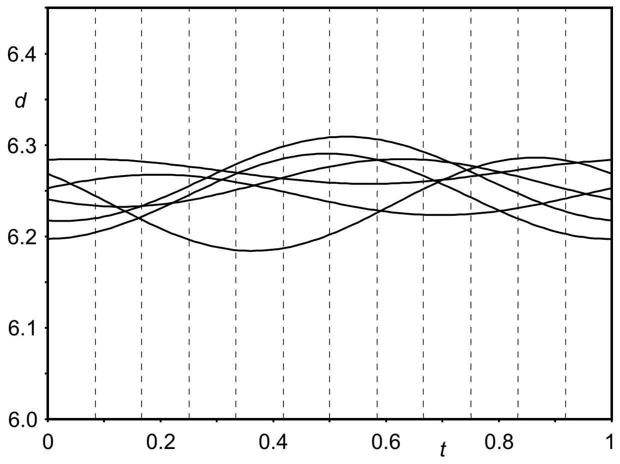

Figure S8: t-Plot of interatomic distances d (Å) between the Co atom and the nitrogen atom of nitrate group C. One cobalt atom is coordinated by six nitrate groups C.

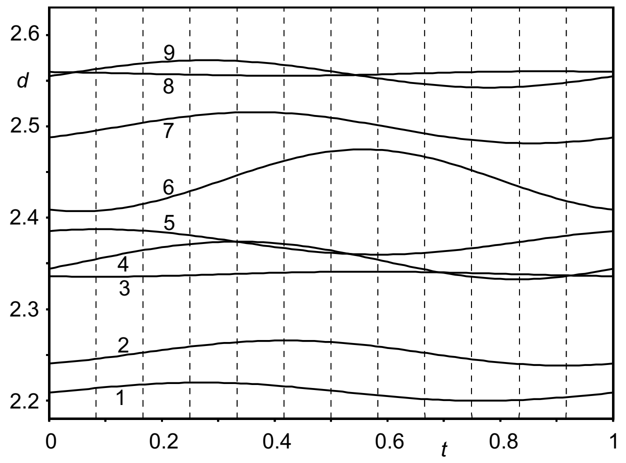

Figure S9: t-Plot of interatomic distances d (Å) between the O atom of nitrate group B and hydrogen atoms of the sepulchrate cage. Vertical, dashed lines indicate t values corresponding to the distances in the 12-fold supercell. Six N–H· · ·O contacts (3, 5–9) and three C–H· · ·O contacts (1, 2 and 4) are shown (compare Fig. 1): 1=O71/3b· · ·H2c36, 2=O71/1b· · ·H2c34, 3=O71/1b· · ·H1n22, 4=O71b· · ·H2c32, 5=O71/3b· · ·H1n22, 6=O71b· · ·H1n26, 7=O71b· · ·H1n24, 8=O71/1b· · ·H1n26, and 9=O71/3b· · ·H1n24. A similar plot for nitrate group A is given in Fig. 9 of the article.

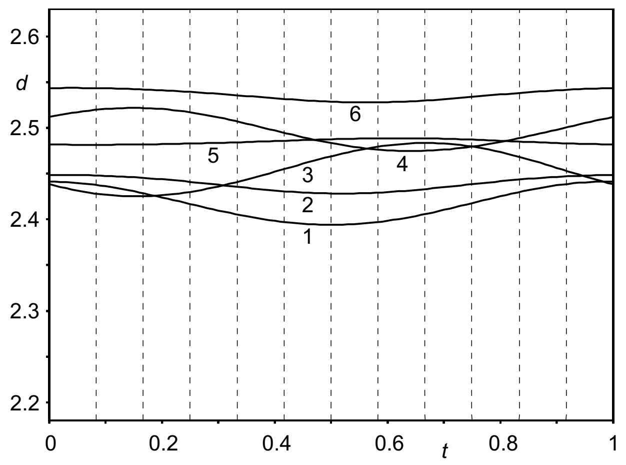

Figure S10: t-Plot of interatomic distances between the hydrogen atoms of carbon atoms C3 on neighboring molecules.

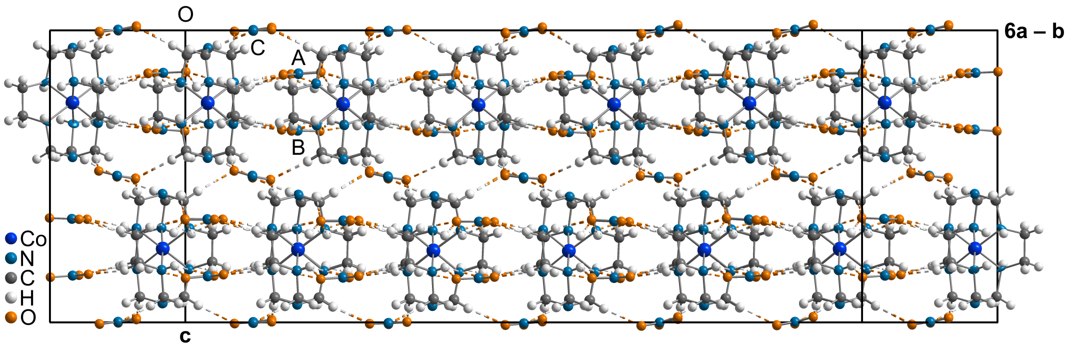

Figure S11: View along (6 1 0) of the supercell of Λ-cobalt(III) sepulchrate trinitrate. N–H· · ·O and C–H· · ·O hydrogen bonds are indicated by dashed lines.

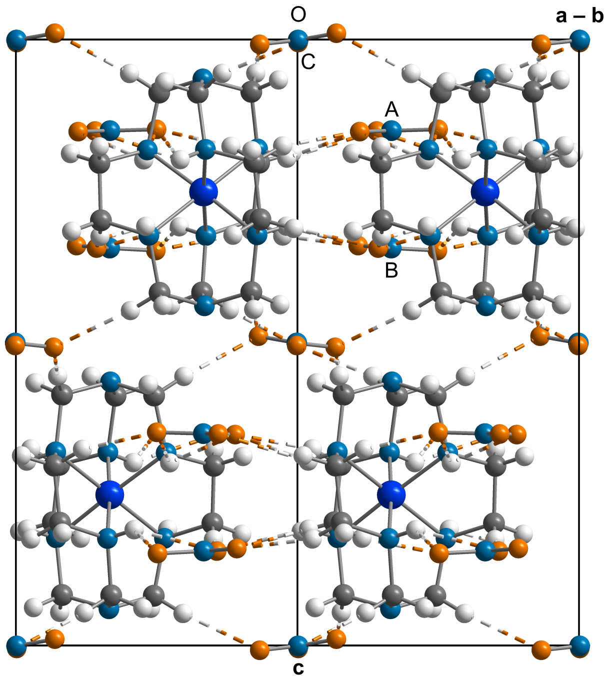

Figure S12: Projection of the crystal structure along (1 1 0) of the basic structure. Dashed lines indicate N–H· · ·O and C–H· · ·O hydrogen bonds of nitrate groups A and B, as well as C–H· · ·O hydrogen bonds involving nitrate group C.

Table S1: Amplitudes of the atomic modulation functions along the three basis vectors. The largest displacements are along **c**; the smallest displacements are along **a**. Magnitudes of displacements are 0.149 Å along **c** for O9 of nitrate group C and up to 0.100 Å for the other atoms; 0.090 Å along **b** for O9 and up to 0.055 Å for the other atoms; and 0.047 Å along **a** for O9 and up to 0.030 Å for the other atoms.

Atom	along **a** (Å)	along **b** (Å)	along **c** (Å)
Co(sep) cage			
Co1	0.0039	0.0209	0.0652
N21	0.0075	0.0314	0.0708
N22	0.0075	0.0368	0.0770
N23	0.0128	0.0332	0.0454
N24	0.0081	0.0300	0.0533
N25	0.0094	0.0288	0.0844
N26	0.0122	0.0330	0.0960
C31	0.0062	0.0226	0.0996
C32	0.0114	0.0381	0.0824
C33	0.0197	0.0277	0.0365
C34	0.0049	0.0259	0.0366
C35	0.0074	0.0185	0.0914
C36	0.0111	0.0266	0.0934
C41	0.0211	0.0607	0.0778
C42	0.0163	0.0500	0.0764
C43	0.0301	0.0409	0.0607
C44	0.0185	0.0538	0.0603
C45	0.0234	0.0396	0.0963
C46	0.0108	0.0540	0.1004
N51	0.0290	0.0545	0.0800
N52	0.0155	0.0548	0.0810
nitrate group A			
N6a	0.0230	0.0408	0.0791
O71a	0.0177	0.0482	0.0658
O71/1a	0.0190	0.0330	0.1056
O71/3a	0.0344	0.0424	0.0845
nitrate group B			
N6b	0.0127	0.0297	0.0709
O71b	0.0147	0.0294	0.0628
O71/1b	0.0156	0.0315	0.0767
O71/3b	0.0077	0.0294	0.0901
nitrate group C			
N8a	0.0283	0.0722	0.0502
O91a	0.0470	0.0897	0.1487
O91/1a	0.0409	0.0634	0.0583
O91/3a	0.0037	0.0645	0.0716
